# South Asian Medicinal Compounds as Modulators of Resistance to Chemotherapy and Radiotherapy

**DOI:** 10.3390/cancers8030032

**Published:** 2016-03-05

**Authors:** N. Rajendra Prasad, Ganesan Muthusamy, Mohana Shanmugam, Suresh V. Ambudkar

**Affiliations:** 1 Department of Biochemistry and Biotechnology, Annamalai University, Annamalainagar Tamilnadu 608002, India; janakganesh@gmail.com (G.M.); mohanabiogene@gmail.com (M.S.); 2 Laboratory of Cell Biology, Center for Cancer Research, National Cancer Institute, NIH, Bethesda, MD 20892, USA

**Keywords:** South Asian plants, ABC transporter, chemoresistance, radioresistance, phytochemicals

## Abstract

Cancer is a hyperproliferative disorder that involves transformation, dysregulation of apoptosis, proliferation, invasion, angiogenesis and metastasis. During the last 30 years, extensive research has revealed much about the biology of cancer. Chemotherapy and radiotherapy are the mainstays of cancer treatment, particularly for patients who do not respond to surgical resection. However, cancer treatment with drugs or radiation is seriously limited by chemoresistance and radioresistance. Various approaches and strategies are employed to overcome resistance to chemotherapy and radiation treatment. Many plant-derived phytochemicals have been investigated for their chemo- and radio-sensitizing properties. The peoples of South Asian countries such as India, Pakistan, Sri Lanka, Nepal, Bangladesh and Bhutan have a large number of medicinal plants from which they produce various pharmacologically potent secondary metabolites. The medicinal properties of these compounds have been extensively investigated and many of them have been found to sensitize cancer cells to chemo- and radio-therapy. This review focuses on the role of South Asian medicinal compounds in chemo- and radio-sensitizing properties in drug- and radio-resistant cancer cells. Also discussed is the role of South Asian medicinal plants in protecting normal cells from radiation, which may be useful during radiotherapy of tumors to spare surrounding normal cells.

## 1. Introduction

The plant kingdom plays an essential role in the life of humans and animals. South Asia is one of the largest producers of medicinal plants and natural products for human health and disease [1]. South Asian countries such as India, Pakistan, Sri Lanka, Nepal, Bangladesh and Bhutan have a large number of plants that are used to produce medicinal agents. In particular, India is one of the largest producers of medicinal plants and can rightly be called the “Botanical Garden of the World”. In India, there are about 400 families and 8000 species of medicinal plants, which include approximately 50% of all the higher flowering plant species [2].

South Asian medicinal plants and their compounds have a very long history of use for cancer treatment. The climate, environment and geography of South Asian countries enable the growth of diverse medicinal plants with novel secondary active compounds, which has attracted the attention of the scientific community. In this review, we focus on the chemosensitizing and radiosensitizing potential of bioactive compounds from South Asian medicinal plants commonly found in Bangladesh, India, Nepal, Pakistan and Sri Lanka, and discuss their mechanisms of action. The chemoprotective properties of South Asian plants have been well documented in the literature [3,4]. The plants and their active compounds intercept carcinogenic pathways at various molecular points and prevent carcinogenesis [5]. Furthermore, South Asian medicinal plants and their compounds have been found to exhibit anticancer properties in different experimental models [6,7]. In fact, anticancer properties of South Asian plants have been recognized for centuries. The active compounds isolated from the plants induce apoptotic signaling in certain cancer cell lines [8,9,10,11,12,13,14,15,16]. For instance, the anticancer property of *Abrus precatorius* has been reported in regard to fibrosarcomas in mice and ascites tumor cells [17]. South Asian plants that have shown anticarcinogenic properties include the anticancer properties of *Albizzia lebbeck* concerning stomach carcinoma in humans [6], *Asparagus racemosa* in human epidermoid carcinoma*, Picrorrhiza kurroa* in hepatic cancers, *Boswellia serrata* in human epidermal carcinoma of the nasopharynx, *Peaderia foetida* in human epidermoid carcinoma of the nasopharynx [18], and *Withania somnifera* in various tumors [19].

Bioactive compounds isolated from South Asian medicinal plants have drawn much attention from researchers, clinicians, and the general public because of their biologic and pharmacologic properties. The plants are excellent sources of macronutrients (carbohydrates, proteins, fats and fiber) and micronutrients (antioxidants, vitamins and trace minerals). In addition, they are the source of an amazing diversity of secondary metabolites (phytochemicals), which are not essential for normal bodily function but are still biologically active and of medicinal value. Phytochemicals found in South Asian plants can be classified into various families such as alkaloids, flavonoids, isoflavones, isothiocyanates, organosulfur compounds, capsaicinoids and phytosterols, which are chiefly found in coloured, leafy vegetables, yellow/orange fruits and some pungent vegetables such as onion and garlic. Many of these compounds have proven chemopreventive properties [20] and their active ingredients are being studied extensively for their antitumor activity. Further, the active components of South Asian medicinal plants possess radioprotective, radiosensitizing and chemosensitizing properties. Phytochemicals such as curcumin, genistein, resveratrol, diallyl sulfide, S-allyl cysteine, allicin, lycopene, capsaicin, diosgenin, [6]-gingerol, ellagic acid, ursolic acid, silymarin, anethol, catechins, eugenol, isoeugenol, isothiocyanates, indole-3-carbinol, isoflavones, phytosterols, folic acid, β-carotene and flavonoids are widely investigated for their radiosensitizing and chemosensitizing properties [21]. Table 1 summarizes the major South Asian medicinal plants and their active ingredients.

## 2. South Asian Medicinal Compounds as Chemosensitizers and Radiosensitizers

Chemotherapy works by targeting cells within the body that divide rapidly, which is one of the prominent characteristics of cancer cells. Radiation treatment damages the DNA of the cancer cells, leading to cell death. One of the primary causes for therapeutic failure in cancer is resistance to radiation and chemotherapeutic drugs. Cancer cells employ several mechanisms to resist chemotherapy and radiotherapy (Table 2). Some tumors develop resistance to structurally and chemically unrelated anticancer drugs, termed multidrug resistance (MDR). This is one of the most dreadful challenges to cancer chemotherapy [22]. Tumors usually consist of a mixed population of malignant cells, some drug-sensitive, and some drug-resistant. Cancer cells that show resistance to chemotherapy when first exposed to an anticancer drug have intrinsic MDR [23]. Chemotherapy usually destroys drug-sensitive cells, and favors the survival of drug-resistant cells. After an initial treatment with chemotherapy, the tumor begins to grow again and develop resistance to chemotherapeutic agents [24]. Furthermore, drug resistance involves altered drug transport across the plasma membrane, genetic responses, enhanced DNA repair, modification of target molecules and access to target cells, metabolic effects, and various growth factors. Some of the mechanisms used by cancer cells to resist cytotoxic drugs are also observed in normal cells. They are part of the defense mechanisms that protect cells from environmental carcinogens.

Multidrug resistance in cancer cells mainly develops by the overexpression of ATP-binding cassette (ABC) drug transporters. This is considered to be a major impediment to clinical cancer chemotherapy [25]. This condition occurs when cancer cells spontaneously become insensitive to structurally and functionally unrelated drugs. ABC transporters utilize energy derived from ATP hydrolysis to actively transport anticancer drugs across biological membranes, preventing drugs from reaching their targets within a cancer cell [26]. The ABC transporters belong to a superfamily of proteins that are classified into seven subfamilies (ABCA-ABCG) based on their sequence. However, only three major ABC drug transporters, including P-glycoprotein (P-gp; ABCB1), multidrug resistance protein 1 (MRP1; ABCC1) and ABCG2 (BCRP; MXR), are believed to seriously affect cancer chemotherapy [27].

Tumor cells acquire changes through many possible mechanisms and have increased resistance to apoptosis. They include inactivation of the p53 tumor-suppressor gene, activating mutations of the gene for PI3K, and activating mutations of the genes for the RAS/RAF pathway. Bcl-2 phosphorylation followed by activation of the NF-κB transcription factor and its downstream targets can lead to resistance to apoptosis. Tumor cell cytokinetics also can contribute to MDR [28], as well as other mechanisms, such as microsatellite instability and defective DNA repair, whereby tumour cells develop genetic changes.

**Figure 1:**
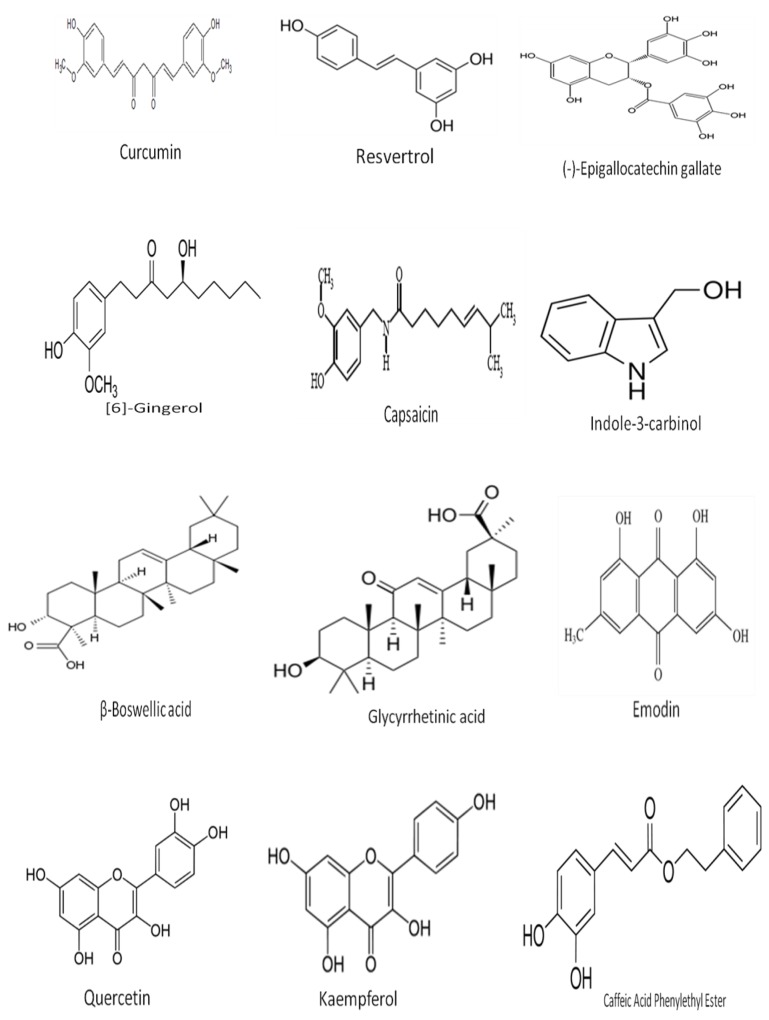
Chemical structure of selected active compounds derived from South Asian Medicinal Plants.

Inhibiting the function of ABC drug transporters by inhibitors or modulators is one method to ameliorate drug sensitivity in multidrug-resistant cancer cells. The inhibition of ABC drug transporters during multidrug-resistant cancer chemotherapy allows elevated drug penetration, distribution and accumulation, and restores drug sensitivity. ABC drug transporter inhibitors, also called MDR modulators, chemosensitizers, or MDR reversal agents, are able to reverse resistance against anticancer drugs [29]. Inhibitors influence ABC transporters by specific interactions with proteins, changing the intracellular ATP level, which is the source of energy, or affecting membrane profiles to increase permeability. Inhibitors can affect other biological targets by nonspecific binding. However, the use of inhibitor drugs at the high concentrations necessary to reach a sufficient inhibitory effect may cause toxicity. Some of the south Asian medicinal compounds commonly used as radiosensitizers and chemosensitizers are listed in Figure 1.

## 3. Phytochemicals as Chemosensitizers

Currently three generations of chemosensitizers have been developed. Unfortunately, they are all toxic and cause side effects in recipients. Phytochemicals from medicinal plants are considered as fourth generation chemosensitizers to overcome tumor resistance. Examples include withaferin-A (from *Withania somnifera*), apigenin, kaempferol, anthocyanidin (from *Solanum nigrum* L); curcumin (from *Curcuma longa*), and berberine (from *Tinospora cordifolia*), glycyrrhizin (from *Glycyrrhiza glabra*) diallyldisulphide, diallyltrisulphide (from *Allium sativum*), and capsaicin (from *Capsicum annuum*). These have been shown to accomplish chemosensitizing activities both *in vitro* and *in vivo* [30]. Curcumin increases intracellular chemotherapeutic drug concentrations of drugs such as vinblastine or vincristine and can inhibit ABC transporters. Curcumin also enhances the cytotoxic effect of 5-FU and sensitizes prostate cancer cells through p53-independent cell-cycle arrest, consequently down-regulating NF-κB activation [31]. Curcumin has been shown to increase the cytotoxic effect of 5-FU and oxaliplatin in colon cancer cells via down-regulation of COX-2 [32,33]. (–)-Epigallocatechin-3-gallate (EGCG) can saturate drug efflux pumps, increasing the accumulation of chemotherapeutic drug within the cell. The substrates in effect sensitize cancer cells to chemotherapeutic agents. Moreover, curcumin is capable of interfering with the function of ABC transporters such as MRP, which needs a balanced supply of reduced glutathione GSH. This kind of inhibition might enhance chemotherapeutic agents by sensitizing cancer cells that overexpress MRP [34]. Targeting c-JUN expression is another clinical strategy to reduce GSH levels to overcome tumor resistance. C-JUN expressions are related with an AP-1–mediated increase in GSH synthetase levels. Curcumin is a strong inhibitor, reducing intracellular GSH at the transcriptional level [35]. Glutathione S-transferase Pi (GST-Pi) expression is linked with resistance to chemotherapeutic agents. Genistein, an isoflavone, is a promising chemosensitizer. Genistein potentiates the efficacy of gemcitabine through the down-regulation of NF-κB and Akt [36,37]; down-regulation of Akt is another mechanism to sensitize cancer cells to the apoptotic effects of TRAIL [38]. Genistein executes its chemosensitizing efficacy by modulating the pathways that cross-talk with TRAIL-induced apoptosis [39]. A number of studies have explored how quercetin enhances the efficiency of anticancer drugs and sensitizes cancer cells to chemotherapy. The efficiency of quercetin to chemosensitize cancer cells to doxorubicin depends on its ability to down-regulate HIF1-α, the downregulation of which makes them sensitive to doxorubicin [40]. Emodin is an anthraquinone obtained from *Cassia obtusifolia*. It is a tyrosine kinase inhibitor that inhibits HER2/neu tyrosine kinase activity, leading to a suppression of tumor growth [41]. Emodin sensitizes chemoresistant cells to paclitaxel-induced apoptosis and increases the intracellular concentration of paclitaxel. It also down-regulates the expression of antiapoptotic molecules and suppresses P-gp expression through ROS generation [42,43,44].

**Figure 2:**
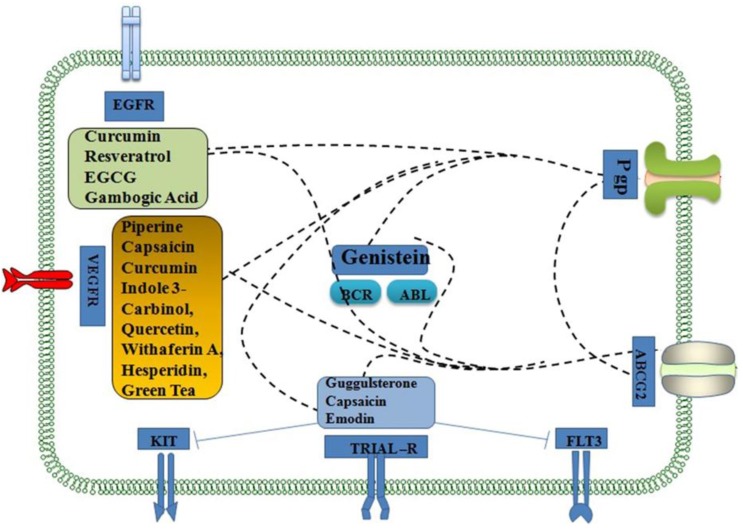
Selected targets of South Asian phytochemicals linked to chemosensitization. The indicated compounds in colored boxes interact with cell surface ABC transporters such as P-gp, ABCG2, and receptor tyrosine kinases including TRIAL-R, FLT3, KIT, VEGFR and EGFR. Some of the medicinal compounds also interact with intracellular tyrosine kinases (BCR-ABL kinase). The dashed lines indicate that the phytochemicals inhibit ABC drug transporters such as P-gp and ABCG2 resulting in chemosensitization. The solid lines depict inhibition of targets such as KIT and FLT3 resulting in chemosensitization.

Resveratrol, a polyphenol, has been shown to sensitize cancer cells to chemotherapeutic drugs by modulating the molecular players of chemoresistance. Resveratrol also increases drug sensitivity through the inhibition of STAT3, NF-κB, c-FLIP, and Bcl-xL [45,46,47]. The major mechanism behind the chemosensitization of resveratrol to paclitaxel chemotherapy is through the downregulation of Bcl-2 family members and P-gp and inhibition of ERK1/2 and AP-1 pathways, leading to reduced Bcl-xL expression in resistant cells [48]. Resveratrol can be effectively used in combination with several chemotherapeutic agents that show adverse effects such as paclitaxel, vincristine, and daunorubicin in some tumor cells [49,50,51,52]. Glycyrrhetinic acid (GA) exhibits cytotoxic activities against HL-60 cells by induction of apoptosis. GA-induced apoptosis is at least partially initiated by interaction of the CD95 and CD178 signaling pathways [53]. Betulinic acid can overcome resistance or cross-resistance effectively. Combination of betulinic acid with irinotecan (IRT) and oxaliplatin (OXT) was effective against SNU-C5/5FU-R cells. Betulinic acid induced cancer cell death by apoptosis through the mitochondrial pathway [54]. The active components of south asian medicinal plants, which have interact with cell surface ABC transporters such as P-gp, ABCG2, and receptor tyrosine kinases including TRIAL-R, FLT3, KIT, VEGFR and EGFR. Some of the medicinal compounds also interact with intracellular tyrosine kinases (BCR-ABL kinase) resulting in chemosensitization (Figure 2).

The ATPase activity of P-gp can be stimulated by number of phytochemicals such as caffeic acid phenyl ester [CAPE], licochalcone A, anacardic acid, celastrol, and xanthohumol. Tumor necrosis factor-α (TNF-α)-mediated NF-κB activation was inhibited by CAPE, licochalcone A, anacardic acid, and xanthohumol. KB/MDR1 cells were found to be sensitized to vinblastine cytotoxicity by CAPE, licochalcone A, anacardic acid, and xanthohumol. Natural NF-κB inhibitors reverse multidrug resistance. Some natural compounds, such as CAPE and licochalcone A, have dual inhibitory effects on the anticancer drug efflux transporter P-gp and NF-κB activation [55]. Natural compounds including withanolide, withaferin A and polyphenol quercetin were shown to inhibit NF-κB target genes in doxorubicin-sensitive K562 and -resistant K562/Adr cells involved in inflammation, angiogenesis, cell cycle, metastasis, anti-apoptosis and multidrug resistance. Withaferin A can overcome attenuated caspase activation and apoptosis in K562/Adr cells, whereas quercetin-dependent caspase activation decreases intracellular protein levels of Bcl2, Bim and P-Bad [56]. The chemosensitizing potential of certain South Asian medicinal compounds are summarized in Table 3.

## 4. Radioresistance

When irradiation is unable to reduce tumor volume or cancer recurs after a regression, then a cell is considered to be radioresistant. Chemotherapy, radiotherapy and surgery are the major therapeutic options for treatment of human malignancies. Nearly 50% of newly diagnosed cancer patients receive radiotherapy (alone or in combination with chemotherapy or surgery) for their treatment [101]. Nevertheless, the curative potential of radiotherapy is limited by intrinsic radioresistance of cancer cells and normal tissue toxicity [102]. The degree of radiosensitivity is recognized by both intrinsic and extrinsic properties. Intrinsic properties include DNA repair, cell cycle status, and survival pathways, while extrinsic properties include cues from the extracellular environment [103]. Radioresistance has an effect on various biological factors such as DNA repair, gene amplification, increases in cellular production of free radical scavengers (eg, glutathione), proto-oncogene activation, and stromal interactions. DNA repair, apoptosis, growth factors, signal transduction, the cell cycle and cell adhesion are associated with genes related to radiosensitivity. The expression of p53 [104], ras [105], raf-1 [106], Bcl-2 [107] and survivin [108] genes are associated with radioresistance. In addition, cancer stem cells have emerged as a contributor to radioresistance through the activation of the DNA damage checkpoint response and an increase in DNA repair capacity [109]. Consequently, there is an increase in interest in enhancing the radiosensitivity of cancer cells for development of effective therapies [110].

## 5. Radiosensitization

A radiosensitizer is a drug that makes tumor cells more sensitive to radiotherapy. Radiosensitization is useful in cancer therapy, as it can enhance the effect of therapeutic radiation. An incredible amount of research and clinical trials have been performed on chemical agents that mimic the radiosensitizing effects of oxygen. Metronidazole, misonidazole, etanidazole and nimorazole are some of the electron-affinity agents used for radiosensitizing effects [111]. Radiosensitizing and radioenhancing agents increase the toxicity of radiation to cancerous tissues, and cause less damage to adjacent normal parenchyma cells. In addition to normal and hypoxia-based radiosensitizers, various other types of sensitizing agents have also been tried on malignant brain tumors. Preclinical research often shows significant promise, but unfortunately, the clinical efficacy of such agents has generally been modest at best [112]. For example, grade III gliomas when treated with conventional radiotherapy and estramustine, an estradiol-based antimicrotubule effector showed a positive improved response. [113].

## 6. Radiosensitizing Properties of South Asian Medicinal Compounds

Radiosensitization has generally been limited to the combination of radiation therapy with traditional cytotoxic agents such as hydroxyurea, 5-fluorouracil and cisplatin. Thus, radiotherapy has been combined with molecular targeted agents, which blocks specific pro-survival signaling pathways [114]. Two brain tumor radiosensitizers have received a great degree of interest of late: motexafin gadolinium (MGd) and efaproxiral [115]. Most patients with cancer receive radiotherapy at some point during the course of their treatment. The efficacy of treatment depends on the total dose of radiation, the fractionation scheme, the degree of oxygenation of tumors and the normal tissue response to radiation. The heterogeneity of tumors and their response to treatment, in early experimental and theoretical studies in radiobiology, were identified. DNA strand-break repair, repopulation of cancer cells between radiation fractions, reoxygenation of tumors after a radiation fraction and redistribution of cells into a radioresistant phase of the cell cycle were considered to be the four classical mechanisms of radioresistance [116]. Understanding the molecular basis of these processes can permit selective targeting of the molecules and pathways that regulate cellular radioresponse. Radiation activates pro-survival signaling pathways, to target normal tissue responses such as angiogenesis and also exploit radiation-induced gene expression for other therapeutic modalities [117].

**Figure 3:**
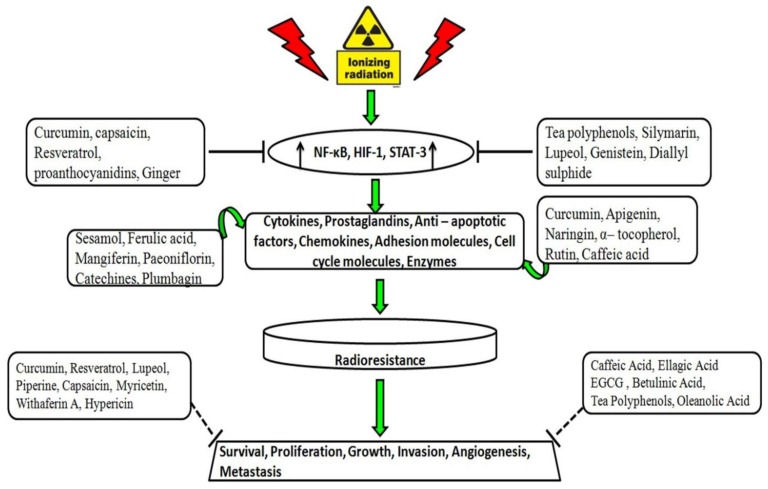
Selected targets of South Asian phytochemicals linked to radiosensitization. Exposure to ionizing radiation leads to activation of several transcription factors, expression of numerous cytokines, adhesion molecules, prostaglandins and enzymes that promote radioresistance. The upward black arrows depict up-regulation of indicated transcription factors. Solid lines indicate South Asian medicinal compounds inhibiting transcription potential of pro-inflammatory transcription factors. The curved green arrows indicate downregulation of expression of proinflammatory cytokines and enzymes involved in radioresistance by South Asian medicinal compounds. Dotted lines indicate South Asian medicinal compounds involved in the inhibition of prosurvival, cell proliferation, growth, invasion, angiogenesis and metastasis through various mechanisms.

Many natural or naturally derived compounds have shown cytotoxic effects in cancer cells. Compounds such as vinblastine, vincristine, taxol, and camptothecin have been approved for clinical practice [118]. These anticancer drugs act through several mechanisms to kill tumors: Microtubule polymerization, mitotic inhibition [119], and inhibition of DNA topoisomerase I enzyme [120]. Genotoxicity is the main tumoricidal effect of anticancer agents. The effectiveness of radiation therapy on cancer cells is enhanced when the anticancer products are co-administered with IR during radiotherapy. Treating cells with radiosensitizers before irradiation probably sensitizes the cell cycle phase to radiotherapy [121,122]. The presence of natural compounds during irradiation enhances their effects by increasing the toxic reactions of free radicals and inhibiting the repair of the radiation induced lethal and sub-lethal damage by misregulation of signal pathways [123]. The active components of south asian medicinal plants, which have rediosensitizing properties, and their molecular targets are described in Figure 3.

## 7. Radioprotective Effects of Natural Compounds

Radioprotectors are agents that protect normal cells during radiotherapy. Radioprotective agents protect macromolecules, cells, and tissues from radiation-induced toxicity. These agents are administered before exposure to radiation. Several mechanisms are proposed for radioprotective agents: Direct scavenging of ROS, hydrogen donation to reactive free radicals, inducing/altering the levels of endogenous enzymes for detoxifying ROS, anti-inflammatory action, immune-stimulant activity, increasing DNA stability, reducing the production of ROS by inducing hypoxia, and enhancing DNA damage repair pathways [124,125,126]. Pretreatment with natural compounds with antioxidant activity protects biological systems against oxidative stress. Because natural products have fewer side effects, they are increasingly being used as radioprotective agents. Polyphenols from herbal plants have protective effects against genotoxicity induced by γ-irradiation. Natural compounds reduce DNA damage and genomic instability induced by ROS *in vitro* and *in vivo*. Flavonoids have excellent antioxidant activity through various mechanisms, and flavonoids with more antioxidant activity have higher radioprotective effects [127]. Table 4 summarizes some of these natural products that act as radioprotectors in normal cells and radiosensitizers in cancer cells through different mechanisms.

## 8. Conclusions

Cancer is a multifactorial disease that requires multichannel therapy, including radiotherapy and chemotherapy. Cancer cells acquire resistance to radiotherapy and chemotherapy through different mechanisms. Number of synthetic and semisynthetic radiosensitizers and chemosensitizers were investigated; unfortunately these compounds exhibit adverse reactions in non-target organs. Naturally occurring medicinal compounds are considered to be non-toxic fourth generation chemosensitizers and a majority of these compounds also exhibit radiosensitizing properties. This present review reveals the role and importance of South Asian medicinal compounds as radiosensitizers and chemosensitizers. South Asian medicinal compounds exhibit radiosensitizing and chemsensitizing property through modulating/inhibiting different molecular targets. Thus, South Asian medicinal compounds may be considered as an adjuvant to radio- and chemotherapy for cancer patients.

## Figures and Tables

**Table 1:** Bioactive compounds from selected South Asian medicinal plants.

Name of the Plant	Bioactive Compounds
*Withania somnifera* L.	WithaferinA, withanolide
*Phyllanthus amarus*	Nirtetralin, niranthrin, phyllanthin, phyltetralin
*Aegiceras corniculatum* L.	Embilins
*Annona muricata*Linn	Annomuricins, bullatacin
*Cedrus deodara*	(−)-Wikstromal, (−)-matairesinoland dibenzylbutyrolactol
*Nothapodytes foetida* Miers	Camptothecin, irinotecan
*Boswellia serrata* Roxb.	Boswellic acid, acetyl-β-boswellic acid, α-pinene
*Andrographis paniculata*	Andrographolide, betulin, betulinic acid
*Centella asiatica* L.	Asiaticoside, hydrocotyline, vallerine, pectic acid, stigmasterol, thankunosides and ascorbic acid
*Coscinium fenestratum*	Berberine, palmatine, 8-oxoprotoberberine, oxypalmatine, berberrubine
*Amoora rohituka* Roxb.	Amooranin, rohitukine
*Trichopus zeylanicus*	Glycophosphosphingolipids
*Bruguiera gymnorrhizais* L.	Diterpenoids and pimaren
*Cuscuta reflexa* Roxb.	Cuscutin, amarbelin, β-sterol, stigmasterol, kaempferol, dulcitol, myricetin, quercetin, coumarin and oleanolic acid
*Dendrophthoe falcata* (L.F.)	Quercetrin, catechin, gallic acid, chebulinic acid, oleonolic acid, β-amyrin-O-acetate, leucocynidin, β-sitosterol and stigmasterol
*Dioscorea bulbifera* L.	Kaempferol- 3,5-dimethyl ether, caryatin, (L)-catechin, myricetin, quercetin-3-O galactopyranoside, myricetin-3-O-galactopyranoside, diosbulbin B
*Embelia ribes Burm.*	Quercitol, diethylnitrosamine/Phenobarbital
*Ficus benghalensis* L.	Lupeol, psoralen and β-sisterol
*Ficus religiosa* L.	Quercetin and qyricetin, stigmasterol and β-sitosterol
*Hibiscus tiliaceus* L.	Stigmasterol β-sitosterol, quercetin, kaempferol
*Jatropha gossypiifolia L.*	Falodone and jatrophone
*Vitex agnus-castus*	Agnucastoside A, agnucastoside B and agnucastoside C, aucubin, agnuside, mussaenosidic acid
*Mollugo pentaphylla* L.	Apigenin and mollupentin, mmollugogenol A, mollugogenol B, mollugogenol D, oleanolic acid and β-sitosterol
*Nelumbo nucifera* Willd.	Liensinine, neferine, pronuciferine, isoliensinine, negferine, asimilobine, nuciferine, remrefidine, isoliensinine, myricetin, quercetin, leucocyanidin, kaempferol, astragalin
*Nyctanthes arbor-tristis* L.	Phenylpropanoid glycosides, carotenoid glucosides, phenyl-propanoid glycoside cardiac glycosides, polysaccharides, β-sitosterol, β-amyrin, hentriacontane benzoic acid, nyctanthic acid, friedelin, lupeol, oleanolic acid, 6β-hydroxylonganin alkaloids, phlobatanins, terpenoids
*Eugenia singampattiana*	4-Hydroxybenzoic acid, caffeic acid, rutin, ferulic acid, coumaric acid, epigallocatechin gallate, quercetin, myricetin, and kaempferol
*Solanum nigrum* L.	Gentisic acid, luteolin, apigenin, kaempferol, m-coumaric acid, anthocyanidin, lunasin
*Strychnos nux-vomica*	Brucine, diaboline
*Zingiber officinale*	Gingerenonea, gingeols, shogaols, zingerone
*Chloroxylon swietenia*	Coumarins xanthyletin, xanthoxyletin and 7-demethylsuberosin
*Podophyllum hexandrum* B	Podophyllin, astragalin
*Linum usitatissimum*	Cynogenetic glycosides
*Glycyrrhiza glabra*	Glycyrrhizin
*Catharanthus roseus*	Vinblastine, vincristine, alstonine, ajmalicine and reserpine
*Camellia sinensis*	Epigallocatechin gallate
*Aloe ferox, Aloe barbadenis*	Aloe-emodin, emodin, aloin acemannan
*Allium sativum*	Alliin, allicin alliin, alliinase, S-allylcysteine, diallyldisulphide, diallyltrisulphide and methylallyltrisuphide.
*Curcuma longa*	Curcumin
*Capsicum annuum*	Capsaicin
*Piper nigrum*	Piperidine, piperine

**Table 2:** Mechanisms by which tumor cells resist radiation and chemotherapeutic drugs.

Radioresistance	Chemoresistance
Tumor hypoxic condition	Increased drug efflux and decreased drug uptake
Increased cellular production of cellular antioxidants	Inactivation of apoptosis
Activation of certain proto-oncogenes, and stromal interactions	Increased drug metabolism and drug compartmentalization
Amplification of DNA repair genes	Increase in the repair of DNA damage
Cancer stem cells as contributors to radioresistance	Increased or altered the drug targets
Survival signals favoured by transcription factors	Survival signals favoured by transcription factors

**Table 3:** Chemosensitizing potential of selected South Asian medicinal compounds.

Natural Products	Anticancer Drugs	Experimental Models	Mechanism of Action	Ref.
Morin	Doxorubicin	MCF-7 MDA435/LCC6 cells	Inhibit P-gp-mediated drug efflux and potentiate doxorubicin cytotoxicity in P-gp positive cells.	[4]
	Vincristine	K562 and K562/ADM	Pentaethylmorin remarkably increased the drug uptake in MDR cells.	[57]
	Daunorubicin	Multidrug resistant human breast cancer cell lines	Increased [3H]daunorubicin accumulation in MDR breast cell lines.	[4]
	Doxorubicin	MCF-7 and MDA435/L	Potentiates doxorubicin cytotoxicity in MDA435/L cells.	[4]
Biochanin A	Doxorubicin	MCF-7 MDA435/LCC6 cells	Biochanin A can potentiate doxorubicin cytotoxicity in Pgp positive cells	[4]
Quercetin	Vincristine	K562 and K562/ADM	Pentamethyl quercetin and pentaallylquercetin remarkably increase drug uptake	[58]
	Vincristine	MBEC4 cells and ddY mice	Increased drug uptake in cells and enhanced brain-to-plasma concentration ratio in mice	[59]
	Doxorubicin	Cultured rat hepatocytes	Reduced drug retention with increase in its efflux	[60]
	Tamoxifen	Female SD rats	AUC, Ka, Cmax increased	[61]
	Paclitaxel	Male SD rats	AUC, Ka, Cmax increased	[62]
	Vinblastine and paclitaxel	MDR KB-V1 cells	Reduced P-gp expression and function.	[63]
Phloretin	Doxorubicin	MCF-7 MDA435/LCC6 cells	Inhibit P-gp-mediated drug efflux; phloretin can potentiate doxorubicin cytotoxicity in P-gp positive cells.	[4]
Nobiletin	Vincristine	K562/ADM	Increased drug uptake in K562/ADM cells.	[57]
Chrysin	Vincristine	MBEC4 cells and ddY mice	Increased drug uptake in cells and enhanced brain-to-plasma concentration ratio in mice	[59]
Kaempferol	Doxorubicin	Cultured rat hepatocytes	Kaempferol potentiated the toxic effect of chemotherapeutic agent and decreasing the efflux of doxorubicin	[64]
(–)-Epigallocatechin gallate (EGCG)	Doxorubicin	P-gp over-expressing KB-C2 cells	Increased drug accumulation	[65,66]
Green tea polyphenols	Vinblastine	Multidrug-resistant cell line CH(R)C5	Potentiates the vinblastine cytotoxicity in CH(R)C5 cells.	[67]
Genistein	Rhodamine 123 and daunorubicin	P-gp-expressing cells	Elevation in intracellular drug accumulation	[68]
Kaempferol	Vinblastine and paclitaxel	MDR KB-V1 cells	Reduced P-gp expression and function	[69,70]
	Tamoxifen	male rats	AUC, Ka, Cmax increased	[71]
Heptamethoxyflavone	vincristine	K562/ADM	Increased uptake of [3H] vincristine	
Phloretin, silymarin	Daunorubicin& doxorubicin	Multidrug resistant human breast cancer cell lines MCF- 7 and MDA435/L	Increased [3 H]Daunomycin accumulation & potentiated doxorubicin Cytotoxicity	[4]
EGCG	Paclitaxel	Breast cancer cells (4T1, MCF-7, and MDA-MB- 231) Female Balb/c mice (4T1) cells xenograft) and various carcinoma cells	Induces apoptosis and increased endoplasmic reticulum chaperone GRP78 expression in tumor tissues. Decreases PCNA immunostaining	[72,73,74]
Curcumin	Carboplatin	NSCLC cell line, A549	Suppression of NF-κB via inhibition of the Akt/IKKα pathway and enhanced ERK1/2 activity	[75,76,77]
	Paclitaxel	Cervical cancer cells	Down-regulation of paclitaxel-induced activation of NF-κB, Akt, and Bcl-2	[78,79]
	Vincristine/ vinblastine	Multidrug resistant KB cells, human multiple myeloma cells	Down-regulated NF-κB or P-gp	[80,81,82]
EGCG + Curcumin	Cisplatin	Ovarian cancer, A2780, A2780cisR and A2780ZD0473R cells	Lower concentrations and shorter time gap between the two treatments produces higher cytotoxic effects	[83,84]
Resveratrol	5-FU	Chemoresistant cholangiocarcinoma tumor model and B16 murine melanoma cells	Down-regulates Cyp1b1 expression and suppresses cell growth and angiogenesis	[85,86]
	Paclitaxel	nonHodgkin’s lymphoma and multiple myeloma cell lines, KBv200	Down-regulation of Bcl-2 family members and MDR1/P-gp. Down-regulation of Bcl-2 family members and MDR1/P-gp.	[48]
	Doxorubicin or vincristine	human uterine cancer cells , doxorubicin-resistant acute myeloid leukemia cells	Down-regulation of MDR1/P-gp and Bcl-2	[87,88]
Caffeic Acid Phenylethyl Ester	Vincristine and Doxorubicine	PL104 cells	CAPE an enhancement of cell death	[89]
Green tea	Doxorubicin	M5076 sarcoma	Increase in accumulation of the antitumor agent	[90,91]
Withaferin A and Siamois	Doxorubicin	K562 and K562/Adr cells	Transcriptional inhibition of NF-κB-, AP1- and Nrf2- and overcome the P-gp-coupled attenuation of caspase-dependent apoptosis in K562/Adr cells	[56]
Piperine	Cyclosproine A	Human colon carcinoma cell line (Caco-2)	Piperine might affect disposition of drugs that are substrates for both P-glycoprotein and CYP3A4	[92]
Capsaicin	5-flourouracil	Gastric cancer cell line HGC-27	Capsaicin has the potential to treat gastric carcinoma with 5-FU *in vitro*	[93]
Diallyltrisulfide	Doxorubicin	K562/A02 cells	Increased expression of Caspase-3 and down-regulation of NF-κB/p65, increasing intracellular adriamycin concentration and inducing apoptosis	[94]
Diallylsulfide	Vinblastine	K562 cells	Enhanced cytotoxic activity of vinblastine as well as other Vinca alkaloids	[95,96]
Emodin	Paclitaxel	MDA-MB-361, MDA-MB-453, BT-483, SKBr-3, and BT-474 cells	Sensitizes HER-2/neu-overexpressing breast cancer cells	[44]
Glycyrrhetinic acid	Daunorubicin	Human carcinoma KB-C2 cells and human MRP1 gene-transfected KB/MRP cells	Dual inhibitory effects on P-glycoprotein and MRP1	[97,98]
(-)-Hydnocarpin	Vincristine	Acute lymphoblastic leukemia cell line	Hydnocarpin potentiating the effect of vincristine in a multidrug-resistant cell line	[99]
Hesperidin	Doxorubicin	MCF-7 cell line, doxorubicin resistant (MCF-7/Dox) cells	Co-chemotherapy application of doxorubicin and hesperidin on MCF-7/Dox cells showed synergism effect through inhibition of Pgp expression.	[100]

**Table 4:** South Asian medicinal compounds protect normal cells from radiation effects and sensitize cancer cells to radiation effects. South Asian medicinal compounds at the concentration studied protects the normal human cells and experimental animals against indicated radiation dose and sensitizes radiation effects in the cancer cells through different mechanism.

Name of the Hytochemicals	Concentration Studied	Radiation Dose	Radioprotective/Radiosensitizing Effect	References
**(i) Radioprotective phytochemicals**				
Curcumin	50 µg/mL	1.5 Gy	Curcumin-encapsulated bioglass-chitosan might have promising potential applications for wound healing resulting from gamma radiation.	[127]
Sesamol	10 μg/mL	4 Gy	(i) Renders protection on γ-radiation induced DNA damage, and antioxidants depletion in cultured human lymphocytes.	[128,129]
	100 mg/kg	7.5 Gy	(ii) Acts as a single prophylactic dose protects hematopoietic and GI systems against γ-radiation-induced injury in mice.	
Ferulic acid	10 μg/mL	4 Gy	Prevents γ-radiation-induced micronuclei and dicentric aberration in human lymphocytes.	[130,131]
	50 mg/kg	4 Gy	Enhances the survival of mice possibly by decreasing DNA damage as examined by γH2AX foci, micronuclei formation, and comet assay.	
Apigenin	10 μg/mL	3 Gy	Significantly reduced (*p* < 0.01) the frequency of mitomycin C-induced micronuclei.	[132]
Mangiferin	5–25 μg/mL	5 Gy	Protects against gamma radiation-induced DNA damage and acts as an antioxidant or pro-oxidant product	[133]
Naringin	50 and 100 µM	6 Gy	Prevents radiation-induced multiple cellular anomalies.	[134]
Paeoniflorin	200 μg/mL	4 Gy	Offers protection against radiation-induced cell damage through modulation of reactive oxygen species and the mitogen-activated protein kinases in thymocytes.	[135]
	50–200 µg/mL	10 Gy	Protected EA.hy926 cells against radiation-induced injury through the Nrf2/HO-1 pathway.	[136]
Luteolin	10 μmol/kg b.wt.	6 Gy	Radioprotective effects through antioxidative property in mice.	[137]
Lignans from *Myristica* fragrans	500 µg/mL	4.26 Gy	Radioprotection through immunomodulation in mammalian splenocytes.	[138]
Hesperidin	50–100 mg/kg b.wt.	5 Gy	Protects against γ-radiation-induced cellular damage and oxidative stress in rats.	[139]
α-tocopherol	360 mg/kg b.wt.	15 Gy	Radioprotective effect of Vitamin E in Parotid Glands in rats.	[140]
Catechines	100 μM	3 Gy	Protects pBR322 DNA under acellular conditions and normal splenocytes under cellular conditions, against γ-radiation-induced damage.	[141]
Orientin	17.5 μM	4 Gy	Promotes stem cell survival, exogenous spleen colony formation (CFU-S).	[142]
Silymarin	50 mg/kg	3 Gy	Protects experimental animals from radiation-induced hepatotoxicity.	[143]
Zingerone	10 μg/mL	2 Gy	Prevents radiation-induced genetic damage and apoptosis in human lymphocytes.	[144]
Rutin	10 mg/kg b.wt.	3 Gy	Mitigates radiation-induced mortality and cytogenetic damage, which attributes to scavenging of radiation-induced free radicals.	[145]
Lycopene	5 mg/kg b.wt.	6 Gy	Protects the small intestine against radiation-induced damage.	[146]
Glycyrrhizic acid	4 mM	1.25 Gy	Offers protection against γ-radiation-induced DNA damage to plasmid pBR322 *in vitro*, human peripheral blood leukocytes and bone marrow cells *in vivo*.	[147]
Naringin	7.5 mg/kg b.wt.	1–5 Gy	Protects mouse bone marrow cells against radiation-induced chromosomal aberrations and lipid peroxidation.	[148]
Quercetin	2–50 μM	30 Gy	Protects against radiation- and storage-induced oxidative damage to RBCs.	[149]
Morin	25 μM	10 Gy	Protects against oxidative stress induced by radiation via reduction of ROS and attenuation of the SEK1-JNK-AP-1 pathway.	[150]
Dehydrozingerone	100 mg/kg b.wt.	10 Gy	Exhibits radioprotective activity in whole body gamma irradiated Swiss albino mice through free radical scavenging. The DMF value was found to be 1.09.	[151]
Famotidine	200 µg/mL	4–12 Gy	Suppresses radiation-induced apoptosis with various doses of gamma-irradiation via radical scavenging and intracellular antioxidation mechanism.	[152]
Pilocarpine	4 mg/kg b.wt.	15 Gy	Amelioration of long term radiation-induced hyposalivation using prophylactic pilocarpine treatment	[153]
Podophyllotoxin	10 nM	10 Gy	Derivatives are used in combination with IR therapy and suggest that the EGFR–p38/ERK–STAT3/CREB-1–EMT pathway might be a useful target for suppressing metastasis.	[154]
**(ii) Radiosensitizing phytochemicals**				
Hypericin	5 µM	8 Gy	Enhancement of radiosensitivity in human malignant glioma cells.	[155]
Epigallocatechin-galate	25 mg/kg b.wt.	22 Gy	Inhibits irradiation-induced pulmonary fibrosis.	[156]
Resveratrol	1 mg/mL	70 kGy	Reduces toxicity and plays a potent role in the treatment of inflammatory disease.	[157]
Curcumin	100 μM	10 Gy	Acts as radiosensitizer through prooxidant mechanisms in cancer cells.	[158]
Gossypol	2 μM	6 Gy	Radiosensitization of tumour cell lines by depressig double-strand break repair mechanism.	[159]
Betulinic acid	20 μM	2 Gy	Induces cytotoxicity and radiosensitivity in glioma cells under hypoxic conditions.	[160]
Plumbagin	750 nM	2 Gy	Radiosensitizing effects in cervical cancer cells through modulation of apoptotic pathway.	[161]
Withaferin A	4 μM	10 Gy	Enhances radiation-induced apoptosis in Caki cells through induction of reactive oxygen species, Bcl-2 downregulation and Akt inhibition.	[162]
Ellagic acid	100 μmol/L	6 Gy	Enhances radiation-induced oxidative stress and subsequent cytotoxicity in tumor cells.	[163]
Caffeic acid	10μmol/kgb.wt.	7 Gy	Exhibits curable effects on gamma irradiation-induced cardiac-oxidative impairment in rats.	[164]
Genistein	20 μM	5 Gy	Acts as a prooxidant in HL-60 cells, increases ionizing radiation-induced cell cycle arrest and sensitivity to apoptotic cell death in human promyeloid leukemia HL-60 cells.	[165]
Myricetin	25 µM	2 Gy	Enhances radiosensitivity of lung cancer A549 and H1299 cells.	[114]
Biochanin A	1–100 µM	2 Gy	Enhances radiotoxicity in colon tumor cells.	[166]
Capsaicin	1–10 µM	1–8 Gy	Increases radiation effects in prostate cancer.	[167]
Piperine	40 μM	15 Gy	Enhances radiosensitivity of tumor cells through oxidative mechanism.	[168]
Lupeol	30 μmol/L	4 Gy	Enhances radiosensitivity of human hepatocellular carcinoma cell line SMMC-7721 *in vitro* and *in vivo.*	[169]
Oleanolic acid	35 µg/mL	250 Gy/min	Radiosensitizes tumor cells through the inhibition of GSH synthesis *in vitro*.	[170]
